# Microplastics in the Environment: Much Ado about Nothing? A Debate

**DOI:** 10.1002/gch2.201900022

**Published:** 2019-06-11

**Authors:** Thomas Backhaus, Martin Wagner

**Affiliations:** ^1^ Department of Biological and Environmental Sciences University of Gothenburg 40530 Gothenburg Sweden; ^2^ Department of Biology Norwegian University of Science and Technology 7491 Trondheim Norway

**Keywords:** evidence‐based policies, microplastics, plastic pollution, precautionary principles, risk assessment

## Abstract

This article documents a debate between the two authors on the issue of microplastics in the environment. It is sparked by a viewpoint published by G. Allen Burton, who argues that the risk of microplastics is overrated. The authors have started debating this notion on Twitter, but the format has quickly turned out to be too cumbersome to exchange arguments. It is thus decided to continue the conversation by exchanging letters published as preprints in roughly four‐week intervals. In these contributions, a broad range of relevant issues are touched upon, including the differences in risk conceptions, risk communication in the attention economy, risk assessment in situations of scientific uncertainty, the need to test proper hypotheses, the problem of prioritizing environmental issues, the costs of action and inaction, the application of the precautionary principle or a strictly evidence‐based approach for policy‐making and, eventually, larger issues related to the Anthropocene. In hindsight, it is felt that this debate is rewarding because it made possible expressing and reflecting on the values and opinions in ways otherwise impossible in social media and standard scientific articles.

## Graphical abstract

**Borne out of an argument over the environmental risks of microplastics** on Twitter, the authors exchange their views on risk conceptions and communication in the attention economy, risk assessment and scientific uncertainty, hypothesis testing, prioritizing environmental issues, the costs of (in)action, the precautionary principle in decision‐making and, eventually, larger Anthropocene issues.

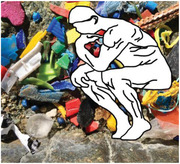

## 1 Introduction

The idea for this slightly unusual article was born from a debate on Twitter. Both authors read the viewpoint article published by G. Allen Burton in Environmental Science and Technology.^1^ In his opinion piece, Burton argues that exposures to microplastics are so low that they do not represent an environmental risk. As a result, Burton concludes that their investigation could be safely abandoned. We both found Alan's text thought provoking but came to different conclusions.

**Figure 1:**
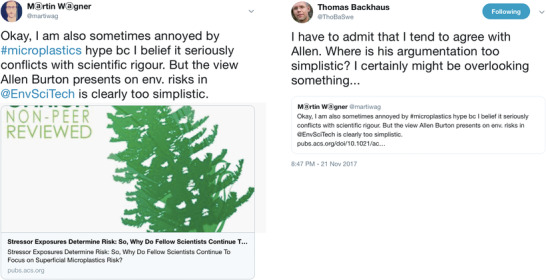
The start of our Twitter debate on the risk of microplastics. The full transcript can be found in the Supporting Information.

Basically, M.W. perceived Burton's viewpoint as “too simplistic,” while T.B. agreed with Burton that the risk of microplastics is overstated (**Figure** **1**). M.W. pointed out that our lack of knowledge on the environmental impacts of microplastics warrants further investigation. T.B. argued that—keeping limited resources in mind—other environmental risks are more pressing than microplastics and deserve our attention. We both agreed that our disciplines are not really good at prioritizing risks and that scientists are too often hunting for the “next big thing” are a result of perverse incentives in academia. The complete Twitter conversation is provided in Table S1 in the Supporting Information.

However, Twitter quickly proved to be too cumbersome for a decent debate. So, we decided to continue the conversation in a format that is more suitable for an exchange of real arguments and viewpoints. This paper documents our conversation, statement by statement.

Our setup for the debate was as follows:

1) An initial statement from M.W.,
2) Comment/rebuttal from T.B.,
3) Response by M.W.,
4) Response by T.B.,
5) Final statement, written independently and in parallel by each author.

Each statement is allowed a maximum length of approximately 1000 words and ten references. A figure counts as 200 words. We have amended this debate article successively with each new letter, publishing updated versions as a preprint.^2^ The final comments were written in parallel by both authors, on the basis of the first four statements.

This paper is, therefore, certainly not a classical scientific article or review paper. Instead, it is an exchange of letters that reflects our individual perspectives, value judgments, and scientific backgrounds. We hope our conversation adds to a broader discourse on the environmental relevance of microplastics.

## 2 Soul‐Searching on Microplastics: Lost in Translation, Prioritization, and Communication?

M.W. (February 27, 2018)

By taking an extreme stance (microplastics = no risk), Burton's polemic^1^ forced me to reflect on my position as well as the underlying arguments and motivations. Sometimes, it takes a devil's advocate to move the debate forward and I am grateful to Burton for playing that role. However, the scientific partisanship regarding microplastics is irritating: In one corner, we have Burton's “null risk” camp directly opposing the “all risk” camp^3^ in the other. Holding extreme positions on either end of the spectrum is valid when backed by strong arguments. I find these missing on both sides (see the Supporting Information). For the sake of this discussion, I will, however, focus on less obvious aspects I encountered during my soul‐searching.

### 2.1 Lost in Translation?

At times, one can get frustrated with sensationalist media reports on (micro)plastics, I give Burton that. The question now is: Does the sensationalism originate in exaggerated scientific publications, as he claims? I believe—for most cases—this is not so. The majority of publications introduce plastic pollution as global problem referring to the massive amount humankind produces and emits. This is something we all can agree upon. They continue by highlighting its potential biological or ecological impacts leading to the specific research question. Although it may become boring reading this over and over again, nothing is wrong with it as long as we take it for what it is: A hypothesis.

Misinterpreting hypotheses as facts is a translational problem, we often encounter in risk communication. Journalists sometimes fall for that fallacy (“microplastics may be toxic” is received as “microplastics are toxic”). Burton does the same when he accuses “fellow scientists” of exaggeration. To test whether this is such misinterpretation, we analyzed the content of microplastics publications in “high‐impact” journals. We found most narratives (66.8%, *n* = 464) on their impacts to be associated with subjunctive phrasing, that is, these indeed are hypotheses (Völker et al.).^4^ Nonetheless, our community can certainly improve in formulating explicit and specific hypotheses to avoid ambiguity. This is something we as authors, reviewers, and editors clearly need to work on.

We encounter another translational issue: As toxicologists, we have internalized a very specific risk conception, namely that risk = exposure × hazard. Other disciplines involved in microplastics research may apply different concepts. For instance, marine biologists often consider microplastics a risk because they are ubiquitous, persistent, and ingested by biota. From this perspective, it is imperative to raise the red flag. Is their risk paradigm less valid than ours? I am not so sure anymore, especially since we have little means to assess the long‐term ecological consequences of (micro)plastics. We might experience “domain inequality” in the sense that one pieces of disciplinary information cannot be understood without completely different expertise.^5^ To solve the wicked problem of plastic pollution, we need to work interdisciplinary. To work interdisciplinary, we need to overcome this inequality and develop a mutual risk understanding.

### 2.2 Attention Deficit Syndrome?

Colleagues often banter about the massive attention microplastics receive, both, inside and outside academia. Rather than culturing professional jealousy, they may worry that the “microplastics hype” withdraws attention and consequently resources from more relevant issues. Although I am not aware that microplastics drain for instance global warming science, this concern reveals a fundamental issue: A system in which researchers vigorously compete for resources produces a range of perverse incentives.^6^ One of the unintended results is that such system rewards those that exaggerate environmental risks. This can even turn into scientific fraud as the recent #perchgate episode painfully demonstrated.^7^

In that sense, we have built a system in which environmental issues compete against each other for attention. This conflict is amplified when it enters the 24/7 news cycle, which favors doomsday communication. Today, microplastics may have won the competition. Tomorrow, there will be another champion (glyphosate, NO*_x_*, etc.). Is this an academic problem? It becomes one once decision makers allocate research dollars according to news coverage. However, we cannot blame others. The root of the problem is rather that the community has no adequate tools to prioritize environmental issues and reach consensus on their relevance. This may be due to the skepticism inherent in the scientific endeavor, disciplinary echo chambers or academic inertia.

In any case, our inability to prioritize diminishes the impact of our science on societies and political decisions. If science cannot decide, societies will decide without science; as the microplastics case illustrates.^8^ If we want our voices to be heard, we should learn from global warming science and instate an Intergovernmental Panel on Chemical Pollution^9^ or on Plastic Pollution for that matter. Such bodies could identify priority pollutants, assess the state of the science and propose research agendas from a multidisciplinary perspective. This would foster building scientific consensus and communicating environmental issues.

### 2.3 Communicating the Right Thing in the Wrong Way?

The aspect I most struggle with is Burton's claim that science has “adversely influenced” political decisions on microplastics. To me, it is obvious that the way we currently produce and use plastics is not only unsustainable but plainly silly. The public debate on microplastics helped exposing the many shortcomings of our linear economy, raised public awareness and generated positive momentum for change. The European Union's Strategy for Plastics in a Circular Economy is one example of this.^10^

Even if the environmental risks of microplastics were low, would we do wrong in promoting a more sustainable use of plastic materials? I do not think so. However, I believe the current narrative we use to legitimize such change is inadequate. It mainly builds on the hypothesized risks of microplastics to wildlife and humans health and often ignores context and uncertainty.^11^ More importantly, a narrative based solely on toxicity neglects other important aspects regarding the societal and economic implications. I believe, we need a new narrative on (micro)plastic pollution that covers all these factors.

## 3 A Genuine Research Topic, But Let Us Avoid Hyperboles

T.B. (March 18, 2018)

It might be useful to frame the topic(s) at hand. There are at least three separate issues, nested into one another:

1) Plastic pollution.
2) Pollution with microplastics.
3) Pollution with microbeads, i.e., deliberately produced microplastic, used in down‐the‐drain personal care products (PCPs).

Plastic pollution is quite obviously a critical environmental problem. For me it falls squarely into the category “so obvious that we need to work on solutions immediately and must not wait for more scientific research,” similar to climate change.

However, microplastic particles (and PCP microbeads in particular) are only small subsets of the bigger problem. Microplastic particles occur globally and are of course a genuine research topic for environmental sciences, including toxicology, ecotoxicology, and risk analysis. But, as M.W. pointed out, we need to be careful about the conclusions we draw, how we move research forward and especially how we communicate the issue to the public and policy makers.

### 3.1 Environmental Risks from Microplastics?

I am squarely with Burton^1^ here. So far, I have not seen evidence that microplastics cause environmental risks, if we define “risk” as a situation where the ratio of exposure and hazard approaches or even exceeds 1. Empirical data and modeling efforts show that microplastic and microbead concentrations are very low in relation to their toxicity to humans and environmental organisms. This seems to hold true not only for direct particle effects but also for effects of microplastic‐associated chemicals.

But maybe I am missing important studies that show otherwise?

No risk being identified at the moment of course does not allow us to conclude that there also will be no risk in the future. Given that in the environment billions of macroplastic items currently disintegrate slowly into microplastic, and in view of the anticipated enormous increase in the production of single use plastics such as packaging,^12^ we will certainly see a massive increase of microplastic pollution in the near future.

### 3.2 Ways Forward

The (eco)toxicological characterization of every conceivable future exposure scenario is impossible, and so is proving the absence of risk. We therefore need to gain a better and more systematic understanding under which circumstances and at which locations environmental risks and risks for human health might develop. We need more and better studies that systematically scan the horizon, contextualize the issues at hand, and finally develop scenarios that guide research and provide policy options.

Especially research on the (eco)toxicology of microplastics seems mainly exploratory at the moment. It is too rarely hypothesis driven and confirmatory (see ref. 13 for a discussion of both research types and their complementary roles in chemical risk assessment and management). We should acknowledge falsifiability as one of the basic principles of scientific inquiry. I would therefore like to suggest the following null hypothesis: *microplastic particles are (eco)toxicologically equivalent to natural organic particles*. Only if we can disprove this initial null hypothesis should we argue that microplastics potentially cause environmental pollution that warrants action.

Working toward falsifying this hypothesis has hands‐down consequences for the design of ecotoxicological studies. In particular, we have to acknowledge the fact that the natural environment is not particle‐free and never has been. Organisms therefore adapt quite well to particles. In an (eco)toxicological study, microplastic‐exposed samples should therefore not be compared to artificially particle‐free controls, but to controls that contain realistic amounts of natural particles.

Microplastics are a case in point that ecotoxicology needs to evolve and become more environmentally realistic, embracing the concept of “stress ecology.”^14^ We need to focus more on ecologically relevant endpoints, and on the role of (micro)plastic in the context of ecological processes and other stressors.

### 3.3 Hyperbolic Statements Have Real‐World Consequences

Environmental research does not happen in a political vacuum. It provides the basis for environmental policy‐making, and it shapes public risk perception. Scientific papers provide implicit or explicit policy advice. I wonder how grossly hyperbolic statements such as “*Microplastic contamination of the oceans is one of the world's most pressing environmental concerns*”^15^ sound to somebody who works in areas devastated by oil production (say, the Gulf of Mexico, the Prince William sound, the Congo basin), or who studies the cooked and bleached corals of the Great Barrier Reef.

It is hardly surprising that such statements from the peer‐reviewed scientific literature (from a *Nature* journal even!) are taken up in new environmental legislation. For example, the current draft of the European Union Directive on the quality of water intended for human consumption reads in its Article 8 on “Hazard assessment of bodies of water:” “*Microplastics are of particular concern due to the negative effects on marine and freshwater environments, aquatic life, biodiversity, and possibly to human health since their small size facilitates uptake and bioaccumulation by organisms, or toxic effects from the complex mixture of chemicals these particles consist of*.”^16^ Where is the evidence that supports such strong statements? At least I could not find any study that demonstrates negative effects of microplastics on, say, biodiversity.

Just like everybody else, I struggle to rank environmental problems. We simply want to tackle them all. But unfortunately, environmental management operates under severe resource restraints and we live in a world with an extremely limited supply of political will and societal motivation to act on environmental issues. So, we have to pick our battles carefully. As the old saying goes: “If they can get you asking the wrong questions, they don't have to worry about answers.”^17^

We have to consider the opportunity costs of hyperbolic statements and the political actions they might trigger: widespread monitoring of microplastics in our water supply, for example, will certainly redirect scarce resources away from monitoring more relevant pollutants. Flagging microplastics as “the most pressing environmental concern” of our times simply trivializes truly critical environmental problems, which are in ample supply.

## 4 Moving Forward: What Are the Risks of Microplastics?

M.W. (April 18, 2018)

There are many aspects in T.B.'s previous statement I fully subscribe to. Importantly, the lack of systematic, conceptual, and hypothesis‐driven research in environmental toxicology is a serious issue, which expands beyond microplastics. While I understand that these shortcomings originate in the history of our discipline, this is something we as a community need to address. This is especially so if we want to evolve from a science tackling very applied problems (e.g., chemical risk assessment) into one addressing more basic questions (e.g., stress biology, to pick up T.B.'s idea). I use the “if” on purpose here because not everybody in the community may share the need for evolving that way.

Take the recent microplastics debate: We are applying a risk framework or conception designed for a very specific application (regulatory decision on the safety of one chemical) to a pretty basic problem (global plastics pollution). To me, this implies that we have decided to frame the plastics problem in a very applied sense, as if we wanted to regulate one compound before it enters the market. As explained elsewhere, I disagree with framing Anthropocene issues in such a narrow way because it neglects the highly interconnected ecological, economic, and societal risks.^8^

Leaving economics and societies aside for a minute, I believe that we have hit a dead end with our reductionist approach to environmental risks. While the classical risk assessment approach has worked reasonably well for chemicals, we now call legacy pollutants, the amount and diversity of synthetic chemicals has tremendously increased since then.^18^ Accordingly, we are not dealing with the “dirty dozen” anymore but with the unknown thousands. In the light of continuing biodiversity loss and assuming chemical pollution is one of its drivers, it is fair to assume that our classical risk framework is insufficiently protective. The reason is simple: It is not built to address the ecological consequences of long‐term exposures to low concentrations of chemical mixtures.

While this problem has been acknowledged by many, I believe we still underestimate the extent by which the traditional PEC/PNEC paradigm has shaped our risk perception and, thus, our research (PEC = predicted environmental concentration, PNEC = predicted no effect concentration). Taking this one step further, insisting on a simplistic, numerical, and almost bureaucratic risk paradigm may be exactly what is preventing us from moving forward, from exploring the idea that risk depends on (ecological) context, from making ecotoxicology more “environmentally realistic.” Again, the microplastics discourse provides the opportunity to critically reflect on our traditional risk paradigm.^8^

My fundamental critique of the PEC/PNEC paradigm notwithstanding, let us view the microplastics problem through that lens: Ignoring information scarcity, methodological limitations, and other uncertainties, we can do the risk assessment exercise for freshwater ecosystems. I use this as a case because toxicological data from “standard” testing are more readily available than for the marine species. Last time I searched Web of Science (27.03.18, search terms “microplastic* AND freshwater AND toxic*”), 27 peer‐reviewed studies were available, including 12 studies actual toxicity studies.

The study reporting the lowest microplastics concentration inducing a significant effect is the one by Wen et al.^19^ Without going into detail, discus fish were exposed to 200 µg polyethylene beads L^−1^ over 30 d. This significantly reduced their predatory performance. Accordingly, the lowest so far reported lowest observed effect concentration (no EC_10_ available) is 200 µg L^−1^ or 871 beads L^−1^ (recalculated). Other than in the usual hazard assessment, I did not evaluate the quality of the study to avoid bias.

Assuming a worst‐case scenario, I looked for the highest reported microplastics concentration in rivers and lakes. For this, I used publications published until 2017 retrieved from Web of Science (search terms “microplastic* AND lake* OR river*” and alternatively “microplastic* AND freshwater”). I screened the results to extract 14 studies reporting actual concentrations in inland waters. Here, the study by Su et al.^20^ conducted on Taihu Lake, China provides the highest so far reported concentrations. Using grab sampling, the authors report a maximum concentration of 25.8 microplastics L^−1^ (PEC). Again, I did not evaluate the study's quality.

If we now apply a low assessment factor of 10 (assuming we had high confidence in the available data), we derive a PNEC for fish of 87 microplastics L^−1^. The risk quotient resulting from the PEC/PNEC ratio is 0.3. Although the margin of safety for the risk quotient to reach 1 ( = risk) is small, we can conclude that at the current date (or better based on current knowledge), microplastics pose no environmental risk according to the traditional approach.

**Figure 2:**
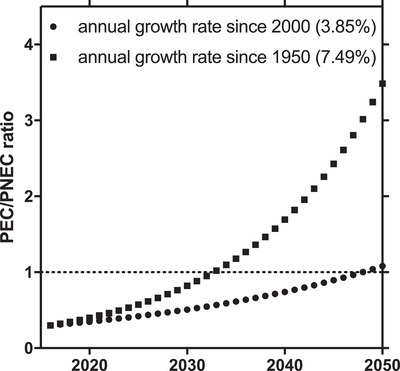
Future projection of the environmental risk of microplastics based on annual growth rates in plastics production (taken from ref. 21). A PEC/PNEC ratio of 1 implies an environmental risk according to the traditional risk assessment framework. This will be reached in 2033 and 2048, respectively.

What about the future? We can perform a prospective risk assessment assuming that no lower PNEC will be established but the production volume of plastics will increase. Using a business‐as‐usual scenario (i.e., no mitigation measures), we can assume increasing production volumes will directly translate to increasing environmental concentrations of microplastics (i.e., PECs). Using the data by Geyer et al.,^21^ the mean annual growth rate of plastics production is 7.48% (since 1950) or more conservatively 3.85% (since 2000). Projecting PEC/PNEC ratios (**Figure** **2**) for these two growth rates results in risk quotients exceeding 1 in the years 2033 (7.48%) or 2048 (3.85%). This leaves us 15–30 years until microplastics would pose an environmental risk according to our traditional assessment framework.

To me, this back‐of‐an‐envelope exercise poses more questions than it provides answers. Provided we insist on framing the risk of microplastics based on PEC/PNEC ratios, we need to ask ourselves:

• How much knowledge do we need to conduct such an assessment?
• How much (un)certainty do we assign to such an assessment?
• Can we apply the approach for the heterogeneous group of microplastics?
• How do we factor in other agents of global change?
• Should we apply the same approach to macroplastics?
• Should we apply the same approach to other Anthropocene issues as well?
• Is the approach adequate for assessing complex environmental issues?
• Is the approach sufficiently protective?
• Do we postpone mitigation actions until the PEC/PNEC ratio reaches 1?
• What will be the costs of inaction?

## 5 We Need to Do a Classical Risk Assessment, but We Cannot Stop There

T.B. (May 18, 2018)

I will split this text into two parts: first a comment on the back‐of‐the‐envelope risk assessment that M.W. presented, and then I will discuss some broader issues on microplastic risk assessment.

### 5.1 A PEC/PNEC Ratio of 0.3?

M.W.'s example resulted in a PEC/PNEC ratio of 0.3, which intrigued me. A margin of safety of not even a factor of four is something that I would consider to be reason for concern. Simply because organisms are not exposed to just that one chemical (or particle), and the “bright line” of a PEC/PNEC ratio of 1 therefore does not provide a sufficient level of protection.

I am more familiar with the (eco)toxicological side of chemical risk assessment, less so with exposure assessments. So, I took a closer look at the study by Wen et al.^19^ Allow me to nit‐pick for a paragraph or two, the study highlights several critical issues.

First of all, the most sensitive organism‐level endpoint used in the study was “postexposure predatory performance,” measured as the number of *Artemia* nauplii (newly born brine shrimps) that a microplastic‐exposed juvenile fish could catch within 5 min. This measurement reflects the ability of a predatory fish to hunt and it was impacted by the microplastic particles.

One would think that an impaired ability to catch food would translate into reduced growth or even death. However, neither was observed, most likely because the fish were supplied with ample food throughout the study. In the end, the authors themselves conclude that “*The results showed that survival rate and body length were not affected by microplastics […]*” and that “*S. aequifasciatus* [the latin name of the investigated fish species] *is prepared to cope with […] microplastics in the water.”*

The study employs, as usual, a control that is completely devoid of any particles. As I have argued previously, this limits the study's relevance. How do we know that natural organic particles, which are found in huge amounts in the fish's natural habitat, would not cause similar effects? It might not be that far‐fetched to assume that a fish needs to put more effort into hunting if it lives in murky waters.

The study investigated just one microplastic concentration (200 µg L^−1^ polyethylene beads), which further limits the risk‐related conclusions that can be drawn. It simply does not allow to draw any conclusion on whether already 201 µg L^−1^ polyethylene beads would be toxic—or whether the fish would happily cope with even 200 000 µg L^−1^.

Every experimental study has limitations. The study by Wen et al.^19^ is a guide for future studies that should use, amongst others, more test concentrations and a more realistic food supply. I certainly like the endpoint “predatory performance,” which strikes me as environmentally very relevant. But in its current form the study does only provide limited information for risk assessment.

### 5.2 Chemical Risk Assessment Is Far from Perfect. But, if Executed Well, It Provides the Basis for the Next Steps

Chemical risk assessment is certainly far from perfect. In particular, it is often hampered by the amount and quality of the underlying exposure and ecotoxicity data. More often than we like to admit, the process merely hobbles along, yielding only semi‐reliable results. However, I would like to point out that the aim of the PEC/PNEC ratio is *not* to provide an absolute risk estimate. It is sufficient if it is protective, i.e., errs on the side of caution.

I would argue that microplastic risk assessment is nothing conceptually new, especially not if we consider the experiences from our research on engineered nanoparticles. Certainly, there are a myriad of technical issues to solve. But are there any reasons to assume that the idea of a risk quotient (i.e., comparing environmental concentrations with a worst‐case ecotoxicological threshold such as the PNEC) would break down if applied to microplastics? If not, then our short‐term goal should be to conduct a series of state‐of‐the‐art risk characterizations, for which we require solid, well documented, and transparent empirical data on exposure and ecotoxicity.

That alone would certainly be insufficient. I wholeheartedly agree with M.W.'s argumentation in the paper by Kramm et al.^8^ The Anthropocene poses challenges for environmental assessments that we cannot ignore. Which brings me directly to his last question: what are the *costs of inaction*? It will be critical to do some more serious forecasting exercises in order to get at least a rough idea on the possible environmental consequences of likely future scenarios.

However, if we indeed look at the issue of microplastics from the perspective of the Anthropocene, that is, from the perspective of a globally interconnected system, one cannot fail to notice that this is only one side of the coin. We also have to consider the *costs of action*. Additional filters for microplastics in sewage treatment plants do not come for free, routine monitoring of microplastics requires a substantial investment of resources that will be lacking elsewhere, and abrasive plastic microbeads for industrial purposes might be replaced with other problematic materials and techniques.

Given the immense global use of plastic, we cannot avoid analyzing the issue in terms of cost‐benefit analyses and comparative assessments. We need to evaluate the societal and environmental costs and benefits of plastic use, we need to optimize its lifecycle and we need to compare it to possible alternatives. In some situations, such as microbeads in cosmetics, this analysis is simple, as the societal benefits approach zero and clearly less problematic alternatives are available. But many scenarios, especially those that involve unintentionally produced microplastics, are far more challenging to evaluate.

In summary, especially from the perspective of the Anthropocene and acknowledging how our political and economic systems currently work, I find it problematic to start implementing specific risk mitigation measures for secondary microplastics, before we do not have at least an indication of an environmental risk. And yes, I am aware that this line of reasoning might indeed run somewhat counter to the precautionary principle. Which is something that I am certainly struggling with.

However, the Rio Declaration (perhaps the most well‐known incarnation of the precautionary principle) begins with “*Where there are threats of serious or irreversible damage lack of full scientific certainty shall not be used as a reason for postponing cost‐effective measures to prevent environmental degradation*.” I guess that brings us back to the question on whether we can come up with a scenario under which microplastic particles could at least hypothetically cause “*serious or irreversible damage*” to the environment or human health.

## 6 Conclusion: To Act, or Not to Act, That Is the Question

M.W. (July 12, 2018)

Arriving at the end of our debate on the risks of microplastics (at least the public part, for now), I realize our positions are neither exclusive nor very controversial: We agree that plastic pollution is a global issue and that scientific standards as well as risk communication regarding microplastics need to be improved. We disagree on the question whether microplastics should be assessed in isolation from or in the larger context of plastic pollution as well as on whether a classical risk assessment approach is appropriate. Because repeating the arguments is not very helpful, agreeing to disagree on those aspects seems sensible to me. However, Pynchon's quote about asking the wrong questions resonated with me. So, I came up with one key question around which, indeed, most of the arguments revolve: To act or not to act on microplastics?

### 6.1 Do We Take a Precautionary or a Strictly Evidence‐Based Approach?

Looking back at our debate, I believe the important question is not so much whether microplastics are a toxicological risk. Most of us can agree that we do not have sufficient knowledge for a meaningful risk assessment yet. The core of the discourse is rather whether to take a precautionary or a strictly evidence‐based approach in terms of risk management, as T.B. highlighted in his previous statement. A precautionary stance legitimates immediate action based on negative impacts of microplastics, which are anticipated but not fully understood based on current scientific evidence. In contrast, a strictly evidence‐based approach aims at reducing these uncertainties to comprehensively understand and assess the problem before taking a risk decision.

There are multiple arguments supporting either approach (see **Table** **1**), but basically, they boil down to the notion that our knowledge on the risks of microplastics is either insufficient (strictly evidence‐based) or sufficient (precautionary). Accordingly, we either need time to understand the problem better before deciding on actions or there are enough reasons to act immediately. The preference for either of the approaches depends on individual values and personalities. Risk‐neutral persons will favor a strictly evidence‐based approach while risk‐averse persons prefer precaution.

**Table 1:** Comparison of a strictly evidence‐based and precautionary approach to microplastics

	Strictly evidence‐based approach	Precautionary approach
Arguments in favor	Insufficient knowledge Low exposure based on current estimates Low toxicity based on current knowledge Presence of “natural” particles at higher levels Likelihood of negative impacts low	Sufficient knowledge Ubiquity Persistence Mobility in the environment and food webs Increasing emissions Is part of macroplastics problem, for which sufficient knowledge on impacts exists Existence of currently unknown, negative impacts
Actions needed	Identify knowledge gaps Perform more research filling these gaps Conduct risk assessment Take risk decision Depending on outcome: develop and implement risk management measures	Take risk decision Develop and implement risk management measures based on fragmentary knowledge Perform research into the effectiveness and efficiency of these measures Refine measures
Advantages	Avoids inefficient risk management measures Avoids unnecessary opportunity and unintended externality costs Avoids regrettable substitutions → Reduce cost of action	Early action avoids negative impacts later Motivates positive societal and economic change (vision of a better society) Fosters technological and societal innovation → Reduce cost of inaction, induce change

There is another layer: When looking at the tentative advantages of each approach, the proponents of a strictly evidence‐based strategy appear to focus largely on avoiding costs of action. In contrast, the supporters of precaution rather want to avoid costs of inaction and promote societal change. From that, we learn that the choice between a precautionary and an evidence‐based approach to microplastics cannot be taken based on science, only. It is also a matter of value judgment (for individuals) and political decision (for societies). Nothing is wrong with that.

The problem is that scientists, policy makers, and other stakeholders are often not very open and explicit about their position on the precautionary principle. For instance, the European Strategy for Plastics in a Circular Economy^10^ does neither refer to the precautionary principle nor to any evidence‐based decision. This dilutes the focus of the debate. So, let me be explicit: Based on my values, I favor a precautionary approach to microplastics, not because I consider them doomsday devices but because I believe in positive change. Microplastics and plastic pollution are the best vehicle we have seen in years to communicate environmental and sustainability issues to the public, engage them in discussions on how we want our future to look like and search for solutions jointly. Continuing this path will certainly involve costs and failure. Nonetheless, I believe the societal and environmental benefits, especially those arising from implementing circularity as guiding principle in our economic system, will eventually prevail.

### 6.2 Does Precaution Mean We Do Not Need Better Scientific Evidence?

Certainly not. The field of microplastics research is young. Accordingly, many studies lack a certain scientific maturity or rigor, even. This is something we need to improve as a community, with journal editors and reviewers playing a key role. More importantly, our research needs to address the important questions. Rather than continuously describing the problem (“we found X microplastics in Y samples”), we need to understand the actual sources of microplastics, the processes driving their fate and the properties driving their impacts. We also need to support the development of better materials, e.g., within the framework of Green Chemistry. This mode of research (a “science of solutions”) will significantly contribute to solving the microplastics issue. Along the road, this approach will solve a range of other issues as well (e.g., that of toxic plastic additives).

### 6.3 Does Precaution Mean We Can Retain the Current Doomsday Communication?

Certainly not. I agree with T.B. that hyperboles regarding the impacts of microplastics are not only inappropriate but also unnecessary. The need for escalation is a feature of the attention economy, researchers should be able and willing to resist. Rather than using fear to attract public attention (or that of funding agencies and editors), we should tell a richer, more complex story, including the moral, social, and economic aspects of plastic pollution and highlight very clearly where knowledge ends and speculation begins. I think that lowering the tone a bit and adding some good old scientific skepticism to the debate will open the road for a conversation we need to have, namely on how to build a sustainable plastics economy.

### 6.4 Does Precaution Mean We Need to Implement All Available Actions against (Micro)plastics?

Certainly not. The Daily Mesh recently mocked the fictional Eleanor Shaw, who “loves lecturing her friends about the evils of single‐use plastic despite the fact that she has a carbon footprint equivalent to a small town in Bangladesh.”^22^ There is some truth in that. We sometimes seem to lose sight of appropriateness in our fight against the plastic tide. Admittedly, I am not a fan of the quick fixes to plastic pollution we are mainly seeing today.

I am not so much worried about economic costs. If these had been significant as Burton^1^ claims, we would have seen a stronger industry backlash. I am not so much worried that the current solutions do not tackle the bulk of the problem. Take the case of plastic straws and the #stopsucking campaign. The initiators acknowledge that avoiding straws will not solve plastic pollution. They rather used it as “gateway plastic” to nudge people to reflect on the larger issue.^23^ Used as vehicle to start a conversation and empower peoples' agency, quick fixes are certainly beneficial.

I am rather worried that in our current mode of solutionism,^24^ the quick fixes on (micro)plastics will cloud our vision of the systemic nature of the problem, namely a linear economy built on consumerism. Systemic problems can only be tackled with systemic solutions. This will need time, a lot of time. I am worried that before we can start working on these, the media and with them the attention of the public and of policy makers will have moved on. So, here is another large question we need to talk about: How do we make the public conversation about environmental issues sustainable?

## 7 Concluding Remarks and a Personal Note

T.B. (July 12, 2018)

The global occurrence of microplastic shows how intimately we live with plastic materials. It is also yet another indicator of how much we, as a species, impact our surrounding. Finding microplastics in every nook and cranny of our planet should therefore make us pause. But, are microplastic particles in and for themselves an environmental problem? What are current environmental impacts, and what are expectable future consequences for ecosystems and for human health?

More and more hard empirical data are emerging on the occurrences of microplastics in marine ecosystems, freshwater, air, and soil. In sharp contrast, empirical data on their toxicity to environmental organisms and to humans remain surprisingly sketchy and almost elusive. Reviews and assessments often merely highlight knowledge gaps and/or speculate on “possible” and “potential” effects. And my apologies for being harsh here, they tend to end up in hyperbolic statements (see also my comments above). To provide yet another example: a recent review by Foley et al.,^25^ summarized that “*Microplastics may pose directly deleterious threat to aquatic organisms worldwide*.” However, the authors admit that “*[…] it could have been insightful to […] examine the relationship between effect size and concentration of microplastics animals were exposed to*.” Indeed. How can one conclude that microplastics are a “*directly deleterious threat*,” if the available data do not allow to relate exposure to toxic effects?

Paracelsus wrote in his third defense that “*all things are poison and nothing (is) without poison. Solely the dose determines that a thing is not a poison*.” If we accept this statement, i.e., that all entities (chemicals or particles) are toxic per se and that the crucial question is by how much we need to lower concentrations in order to reach a “safe” level, we have to draw at least four conclusions: 1) microplastic assessments need to be based on data that describe the quantitative relationship between microplastic exposure and toxic effects, and consequently 2) toxicity studies have to be better designed, and be based on testing whole concentrations series, including environmentally relevant particle densities. 3) Conclusions need to be drawn from a comparison with relevant controls (see above) and finally (4) findings of “no effects” need to be as systematically published as findings of toxic effects.

Even if they are of good quality, empirical data will always have limitations. Chemicals with PBT or vPvB properties (Persistent, Bioaccumulative and Toxic, and very Persistent and very Bioaccumulative, respectively) therefore warrant special attention, as they spread globally and cannot be managed after they have been emitted into the environment. Such chemicals therefore do not provide any opportunity to correct erroneous decisions of the past. Microplastics are sometimes equated with such compounds, e.g., with polychlorinated biphenyls (PCBs).^26^

To me this is a false equivalency. First, several PCBs are, in contrast to microplastics, potent endocrine disrupters. Second, microplastics do hardly, if at all, bioaccumulate or biomagnify—certainly not to the same extent as PBT/vPvB chemicals. Although studies have managed, after painstaking efforts, to find some microplastic particles embedded in biological tissue and transferred through the food chain, it is still more than a far stretch to equate these particles with PCBs, brominated flame retardants, dioxins, and similar compounds, who are found in organisms in concentrations thousands of times higher than in the surrounding environment.

### 7.1 The Broader Picture

The global occurrence of microplastics forces us to (re‐)examine some fundamental issues. Several are almost evergreens, especially for environmental scientists and sustainability experts. But it might still be worth revisiting them in the context of the (micro)plastic issue.

1) 7.6 billion people with a constantly increasing per‐capita resource consumption^27^ will unavoidably leave their mark on the planet. The wicked challenge is to differentiate between acceptable and unacceptable impacts, which is obviously a highly context‐dependent societal value judgment.

So, we need to take a hard look at the societal benefits (or lack thereof) of plastic products. Sometimes there is widespread agreement and/or nonplastic alternatives exist, which is why banning drinking straws, plastic cutlery, and microbeads from cosmetics is easy. But when it comes for example to plastic food packaging, things might not be as simple. In the end, the challenge will be to find a better balance. The Swedish word “lagom” (“just about right”) comes to mind.

2) Given that we are living in an increasingly busy world, more and more decisions will have to be a selection between competing alternatives. That is, in the future we will not often have the luxury to simply consider the costs of inaction. We will also have to consider the costs of noninaction and its (perhaps unintended) consequences. As outlined above, I argue that prematurely acting on the mere occurrence of microplastics in the environment derails societal resources from more pressing matters.
3) We lack a system of planetary governance, despite the increasing realization that we live in the age of the Anthropocene. In principle, we could learn from the Montreal Protocol (which aims to protect the stratospheric ozone layer by phasing out the production and consumption of ozone‐depleting substances) or the Rotterdam convention (the informed consent procedure for hazardous chemicals and pesticides in international trade). Both are quite successful global environmental agreements and we could use the collated experience to establish global best practices for plastic production, trade, use, and recycling. We could even consider caps and bans on the production of certain plastic items.

Unfortunately, the current global tendency toward populistic, short‐sighted “country first” politics provides little ground for optimism. I doubt that we can expect to see a global environmental agreement on plastic coming to life any time soon.

4) Increasing chemical and plastic production is widely regarded as positive, given that such industries create jobs and that many of the produced materials tremendously increase the quality of human life. In many places, the downside of these trends is only slowly realized. As scientists, especially as academics, I would argue that it is our obligation to get involved in the ensuing societal debates, in order to help exploring options and scenarios with the aim to contribute to a better understanding of the consequences of political and societal (in)action.^13^, ^28^
5) Science must be broad and environmental science must explore issues on the far horizon. Otherwise we will not have the canaries to put in our coalmines, and we will not have science‐based policy options to steer future developments. Consequently, science (in particular environmental science!) must also explore issues that do not have direct, obvious policy relevance. Unfortunately, this runs counter to the current trend to assess scientific work, especially in the environmental sciences, almost exclusively in terms of its short‐term usefulness—and to steer funding disproportionally toward this type of work.

Contrary to science, politicians and decision makers have to prioritize more intensely, given the limited availability of resources and fickle public attention. A functional bidirectional science‐policy interface is therefore needed. For this purpose, Europe has established a dedicated Science Advice Mechanism for the European Commission (https://ec.europa.eu/research/sam/index.cfm) and the Science Advice for Policy by European Academies (https://www.allea.org/asap-academies-sciences-advice-to-policy/) project. On the international level, the Strategic Approach to International Chemical Management (https://www.saicm.org/) that is hosted by UN Environment is discussing similar activities for its post‐2020 work.^29^

In summary, I would submit that microplastic in the environment is an issue certainly worthy of scientific investigation. In that aspect, I disagree with Burton's^1^ point of view. But, to me, it is not an issue that warrants political or societal action just right now. Not before we do not have a better understanding of how we could/should act.

This should certainly not be taken as a call to “wait and see” with respect to the broader issue of plastic pollution. Curbing rampant plastic overconsumption, which is all too often paired with woefully inadequate waste management, is a global task at hand right now, for a whole variety of reasons. And we know pretty well what we would need to do. The only question is whether we, as a global society, will muster the political and societal will to actually get going.

### 7.2 A Personal Note at the End

This conversation has been rewarding and I definitely learned a thing or two or three. Hopefully, we will be able to continue the debate elsewhere. With more than 2000 views and more than 1000 downloads of the preprint even prior to our concluding remarks, it feels as if we even have some readers that are interested in this format and/or in the topic of discussion.

So, I certainly owe M.W. sincere thanks for taking time out of a busy schedule in order to participate in this experiment, and for daring to engage in a public “soul‐searching” on a research topic close to his heart!

## Conflict of Interest

M.W. is an unpaid member of the Scientific Advisory Board of the Food Packaging Forum (FPF). M.W. does not receive any personal benefits from this work, financial or otherwise. T.B. is a board member of the FPF and the International Panel on Chemical Pollution (IPCP), two NGOs that work on the issue of plastic packaging and chemical pollution in general. T.B. does not receive any personal benefits from this work, financial or otherwise.

## Supporting information

Supporting Information

## References

[1] G. A. Burton Jr. , Environ. Sci. Technol. 2017, 51, 13515. 29148729 10.1021/acs.est.7b05463

[2] T. Backhaus , M. Wagner , peerJ Prepr. 2018, 6, e26507v6.

[3] C. M. Rochman , S. M. Kross , J. B. Armstrong , M. T. Bogan , E. S. Darling , S. J. Green , A. R. Smyth , D. Verissimo , Environ. Sci. Technol. 2015, 49, 10759. 26334581 10.1021/acs.est.5b03909

[4] C. Völker , J. Kramm , M. Wagner , On the Creation of Risk: Framing of Microplastics Risks in Science and Media. Global Challenges 2019, 1900010, 10.1002/gch2.201900010.

[5] Nature 2017, 552, 148, 10.1038/d41586-017-08465-1. 29239392

[6] M. A. Edwards , S. Roy , Environ. Eng. Sci. 2017, 34, 51. 28115824 10.1089/ees.2016.0223 PMC5206685

[7] M. Enserink , Science 2017, 358, 1367. 29242324 10.1126/science.358.6369.1367

[8] J. Kramm , C. Volker , M. Wagner , Environ. Sci. Technol. 2018, 52, 3336. 29494144 10.1021/acs.est.8b00790

[9] M. Scheringer , Chemosphere 2007, 67, 1682. 17207837 10.1016/j.chemosphere.2006.11.023

[10] European Commission , https://ec.europa.eu/environment/circular-economy/pdf/plastics-strategy.pdf (accessed: February 2018).

[11] S. Rist , B. Carney Almroth , N. B. Hartmann , T. M. Karlsson , Sci. Total Environ. 2018, 626, 720. 29396337 10.1016/j.scitotenv.2018.01.092

[12] The Guardian , https://www.theguardian.com/environment/2017/dec/26/180bn-investment-in-plastic-factories-feeds-global-packaging-binge (accessed: March 2018).

[13] T. Backhaus , X. Trier , Integr. Environ. Assess. Manage. 2015, 11, 183. 10.1002/ieam.1630 25820305

[14] N. Van Straalen , Environ. Sci. Technol. 2003, 37, 324A. 10.1021/es0325720 12967088

[15] R. Hurley , J. Woodward , J. J. Rothwell , Nat. Geosci. 2018, 11, 251.

[16] European Commission , COM(2017) 753 Final, Brussels, 2017, https://eur-lex.europa.eu/resource.html?uri=cellar:8c5065b2-074f-11e8-b8f5-01aa75ed71a1.0016.02/DOC_1%26format=PDF.

[17] T. Pynchon , Gravity's Rainbow, Viking Press, New York, NY 1973.

[18] E. S. Bernhardt , E. J. Rosi , M. O. Gessner , Front. Ecol. Environ. 2017, 15, 84.

[19] B. Wen , N. Zhang , S. R. Jin , Z. Z. Chen , J. Z. Gao , Y. Liu , H. P. Liu , Z. Xu , Aquat. Toxicol. 2018, 195, 67. 29288934 10.1016/j.aquatox.2017.12.010

[20] L. Su , Y. Xue , L. Li , D. Yang , P. Kolandhasamy , D. Li , H. Shi , Environ. Pollut. 2016, 216, 711. 27381875 10.1016/j.envpol.2016.06.036

[21] R. Geyer , J. R. Jambeck , K. L. Law , Sci. Adv. 2017, 3, e1700782. 28776036 10.1126/sciadv.1700782 PMC5517107

[22] The Daily Mesh , https://www.thedailymash.co.uk/news/environment/woman-who-makes-huge-fking-deal-about-plastic-straws-always-flying-everywhere-20180629174770 (accessed: July 2018).

[23] The Gateway Plastic, https://www.globalwildlife.org/2017/10/19/the-gateway-plastic (accessed: July 2018).

[24] E. Morozov , To Save Everything, Click Here: The Folly of Technological Solutionism, PublicAffairs, New York, NY 2013.

[25] C. J. Foley , Z. S. Feiner , T. D. Malinich , T. O. Hook , Sci. Total Environ. 2018, 631–632, 550. 10.1016/j.scitotenv.2018.03.046 29529442

[26] C. M. Rochman , Science 2018, 360, 28. 29622640 10.1126/science.aar7734

[27] International Resource Panel , U. N. Environ. Programme, Nairobi, Kenya 2017, https://www.resourcepanel.org/sites/default/files/documents/document/media/assessing_global_resource_use_amended_130318.pdf.

[28] S. E. Apitz , T. Backhaus , P. M. Chapman , W. Landis , G. Suter , Integr. Environ. Assess. Manage. 2017, 13, 557. 10.1002/ieam.1937 28613028

[29] T. Backhaus , M. Scheringer , Z. Y. Wang , Integr. Environ. Assess. Manage. 2018, 14, 432. 10.1002/ieam.4052 29906353

