# Supplementary material for: Microplastics in the Environment: Much Ado about Nothing? A Debate
Source: Glob Chall. 2019 Jun 11;4(6):1900022. doi: 10.1002/gch2.201900022 (PMC7268194; doi:10.1002/gch2.201900022)
Supplement: Supplementary file 1 — Supporting Information [file GCH2-4-1900022-s001.pdf]

Copyright WILEY-VCH Verlag GmbH & Co. KGaA, 69469 Weinheim, Germany, 2020.

Supporting Information

for *Global Challenges,* DOI: 10.1002/gch2.201900022

Microplastics in the Environment: Much Ado about Nothing? A Debate

Thomas Backhaus and Martin Wagner**

Copyright WILEY-VCH Verlag GmbH & Co. KGaA, 69469 Weinheim, Germany, 2019.

Supporting Information

Microplastics in the environment: much ado about nothing? A debate

Thomas Backhaus, Martin Wagner**

Table S1: Word-by-word transcript of the authors' debate on Twitter, conducted on Nov 21, 2017.

Martin Wagner (@MartiWag)	Thomas Backhaus (@ThoBaSwe)
Okay, I am also sometimes annoyed by #microplastics hype bc I belief it seriously conflicts with scientific rigour. But the view Allen Burton presents on env. risks in @EnvSciTech is clearly too simplistic. https://pubs.acs.org/doi/10.1021/acs.est.7b05463	
	I have to admit that I tend to agree with Allen. Where is his argumentation too simplistic? I certainly might be overlooking something...
I/II: In 280 chars: In his piece, Burton<br>- assumes low exposure based on studies of very large MP not covering relevant sizes<br>- assumes no hazard based on handful of tox studies conducted mainly with PS beads	

Martin Wagner (@MartiWag)	Thomas Backhaus (@ThoBaSwe)
Bottom line: Does risk assessment in absence of required knowledge	
	Well, he does not really do a risk assessment. But he argues that we know that there are massive ecosystem-wide risks, which we should study first. Unless we have at least an idea when/how/where MP could actually have env. impacts. Do we have that (honest question)?
Well, he claims no risk throughout the piece. Leaving this aside, #microplastics are just another aspect of #globalchange that we need to look out for. When, where and if there will be risk I cannot even best guess today. Does that mean we should stop investigating? #Idontthinkso	
	That's an interesting question... In a world with sufficient resources for env research: certainly not. In the current situation: maybe we should focus our time, effort and resources on more pressing matters?
Otherwise, I agree with him that authors, reviewers and editors need to raise the bar, resist the hype and significantly increase quality of #microplastics research.	
	The statement that I actually don't agree with, is the notion that we should not bother to limit unnecessary env. exposures, unless we already know they're causing harm (and not a minute earlier). #precautionaryprinciple
Indeed, waiting for "final proof" (which does not exist anyway) may be missing the last bus. Plus: Societies and politics have decided to act on #plasticpollution already. W/o consulting us. May be blow to scientist's ego but we need to accept and work on what they task us with.	
	#plasticpollution is an absolutely critical environmental issue, no doubt. I think we completely agree on that. But are #microplastics ?

Martin Wagner (@MartiWag)	Thomas Backhaus (@ThoBaSwe)
	Btw, I hope you're right in your assessment that society and politics actually DO start to act.
No either or here: #plasticpollution and #microplastics are the same. Just different in size. Problem is that we cannot dump #macroplastics on whales in the lab.<br>And different in numbers (I expect exponentially more nano than micro than macro).	
	But I actually beg to differ. There's clearly proven harm caused by macroplastics to macrofauna. And it's completely reshaping ecosystems (an effect of which we know far too little).
II/II: What I find simplistic is that Burton treats MP as one entity whereas in fact it is a huge group of potential stressors. Furthermore, his view is solely thu ecotox lens, ignoring wider ecological & societal implications of #plasticpollution. We must be more holistic here.	
	In all fairness, Allen doesn't touch upon the general issue of #plasticpollution Which might be a shortcoming of the paper, indeed. But being holistic would also imply to realize where the big issues are, not re-arranging deckchairs on the Titanic.
Sure. Big issues are #consumerism and linear economy. Unfamiliar deckchairs for ecotoxicologist to sit on, I admit. But either we can stay seated or help to redesign...	
	Yeah, let's go down into the engine room! But even from an ecotox perspective... perhaps there are more pressing issues to explore?<br>I'm not saying that #microplastic research is pointless. But more critical distance would sometimes be good.
First, I'm not convinced our discipline has managed to prioritize very well. Second, I belief everybody going after the one ring is not how we should approach #globalchange. Third, this is especially true for #microplastics. Diversification needed. Hampered by funding practice.	

Martin Wagner (@MartiWag)	Thomas Backhaus (@ThoBaSwe)
	No, we're lousy at prioritizing. That's basically Allen's point, isn't it? And there are myriads of "rings" out there that need urgent attention... What kind of diversification would you like to see in #microplastic research? Or better: where do you see the critical issues?
At this point: Start determining env. conc. of #microplastics < 300 µm (preferably down to 2 µm, which is technically feasible) and you will find tons.	
	...in what volumes of water / sediment? And, even if so, does it matter - or is it just another type of organic particle?<br>Heretical questions, I know. Sorry...
For which you will burn on a pile of plastic waste, of course. In terms of scepticism I am with you. In terms of closing the case not. Rather want to know more before moving on to the #nextbigthing.	
	Maybe that's what I'm arguing for: let's limit the hunt for the #nextbigthing a bit. Instead let's do more solid, bread-and-butter, middle-of-the-road research instead.<br>But yeah, that wouldn't sit well with a lot of funding agencies... Unfortunately.
Absolutely, perverse incentives in academia is something that needs addressing as urgent as #plasticpollution.<br>http://online.liebertpub.com/doi/10.1089/ees.2016.0223	
	Yepp, I was just about to send a link to that paper also...!
